# Labyrinthitis

**Published:** 1924-07

**Authors:** E. Watson-Williams

**Affiliations:** Teacher of Laryngology, University of Bristol


### LABYRINTHITIS.

### Remarks, with Special Reference to Treatment, on Cases shown to the Bristol Medico-Chirurgical Society, May 14th, 1924.

BY

E. Watson-Williams, M.C., Ch.M.,

*Teacher of Laryngology, University of Bristol.*

---

Evidence of labyrinthine disturbance in the course of acute or chronic middle-ear disease, catarrhal as well as suppurative, is by no means rare. An actual labyrinthine lesion is a much less frequent complication of aural suppuration; but this last condition is so common that the numbers of sufferers from labyrinthitis—placed in one series of statistics as high as ten per cent. of all cases of otorrhœa—must be considerable.

It is not the purpose of this communication to discuss the somewhat special subject of the diagnosis of labyrinthine disease. The manifestations may vary from the very slightest to the most severe and alarming. At either end of the scale nausea or vomiting may attract more attention than the vertigo, nystagmus, etc., which one is inclined to regard as characteristic of labyrinthitis. Many patients who do not spontaneously complain of vertigo say that they can never take more in the morning than a cup of tea; they do not associate this slight nausea with any aural trouble. On the other hand, acute labyrinthitis may render the patient so ill that his vertigo does not attract notice; fever, prostration and frequent vomiting lead to the mistaken diagnosis of gastric influenza. Between these extremes giddiness is the common complaint, and may vary from unsteadiness on stooping or on exertion to attacks in which the sufferer falls to the ground. Varying degrees of ataxia may also be seen, *e.g.* the patient may complain that in hammering he frequently misses the nail and hits his left hand ; or he may find that in walking he must always keep to one side of a companion—if he tries the other he constantly jostles him. In the presence of middle-ear disease, even though the latter is of old standing and apparently unchanged, disturbances of the stomach or of equilibrium (in its widest sense) demand the consideration of a possible connection.

A word may be said of one sign of labyrinthine disease, the FISTULA SIGN. As originally described by Barany it is as follows : When the air pressure in the meatus is raised there is *instant* deviation of the eyes to one side (commonly the sound side) and nystagmus to the other side ; when the pressure is reduced there is *instant* reversal of the phenomenon. The nystagmus stops abruptly on releasing the pressure or suction. A number of other tests are described, but this is the only one I have felt really indicative of a fistula. The sign is not very common ; to elicit it only gentle pressure is required ; instant response, and instant reversal, are essential points. More marked pressure will in a large number (? 50 per cent.) of cases with otorrhœa give a similar sign after a latent period of two to five seconds. This is the " PSEUDO-FISTULA " sign, and its usual direction is the reverse of that most common with the true fistula sign. It does not indicate labyrinthine disease. I have even met with this phenomenon as a symptom. The patient, a builder, was the subject of right chronic suppurative otitis media, without any labyrinthine lesion ; the fistula test was negative on many trials. Recounting his symptoms, this man said, " If I put the wool into my ear too hard everything goes round to the left ; but if I pull it out too sharp everything goes round to the right." Needless to say, either form of response is indicative of a living labyrinth, and may sometimes even be obtained when the labyrinth does not respond to the caloric test.

Otogenic labyrinthitis may be acute or chronic ; it may further involve the whole labyrinth, or a part only. In the latter case—circumscribed labyrinthitis—the labyrinth will respond to some of the tests of function ; in the former—pan-labyrinthitis—there will be no response, except perhaps during the first five or six hours. The distinction has been made between acute serous and acute suppurative pan-labyrinthitis ; the diagnosis rests on whether there is or is not eventually some restoration of function, and clinically can hardly be made in the acute stage. Further, acute labyrinthitis may supervene on acute or on chronic middle-ear disease, or on chronic circumscribed labyrinthitis ; chronic labyrinthitis may follow an acute attack, or may be chronic *ab initio ;* indeed, cases have been described where the entire labyrinth is destroyed, but so insidiously that labyrinthine symptoms have never attracted attention. The severity of symptoms is related to the rapidity of destruction of function, rather than simply the extent of such destruction.

The correct diagnosis of any given case has an important bearing on the treatment. When should one operate on the labyrinth ? One class of case imperatively demands such an operation. When acute pan-labyrinthitis supervenes on a chronic otitis media, or on chronic circumscribed labyrinthitis, the labyrinth soon ceases to respond to tests. It is, of course, possible that the damage will be arrested naturally ; unfortunately, if it is not, since the labyrinth gives no indication of what is occurring, the next signs of progress of the disease are those of suppurative meningitis. Some authorities even consider the majority of all cases of septic meningitis as due to labyrinthine infection. It is true that even where meningitis is present the situation is not quite hopeless; but the chances of recovery have been gravely jeopardised. If, therefore, such a case is seen early, the labyrinth should be drained.

**Case 1.**[1] Acute suppurative pan-labyrinthitis, complicating chronic circumscribed labyrinthitis. Operation, inferior vestibulotomy. Recovery, with relief of previous vertigo.

W. A. B., male, aged 40, labourer.

November, 1923, c.c.o. deafness and discharge from right ear, and vertigo since 1918, when a radical mastoid operation had been performed (apparently for labyrinthine symptoms). The attacks were not severe, two or three a week. Auditory bone conduction was present, but diminished; labyrinth responded to tests; never vomiting. Treatment greatly improved the condition.

At the end of February, 1924, he had an attack of vertigo very much more severe than ever before, with increase of discharge and pain in the ear. There was complete prostration, with much vomiting. Seen the next day he was still very ill, unable to walk alone. Temp. 100° F. Severe vertigo, spontaneous nystagmus (mixed) to the left. Ear deaf to tuning forks, labyrinthine tests entirely negative. He was admitted at once to hospital, and I curetted out the vestibular contents. He made a good recovery, and is now free from all vertigo.

If the case is not seen within the first three days—the prognosis gets steadily worse hour by hour—different considerations arise. Either signs of meningitis will be appearing, or there is some hope that the infective invasion has been arrested. In the first case, surgical measures outside the scope of this paper may be proper; in the second, we may congratulate our patient on a very fortunate issue. When the labyrinth is dead, either no operation should be done; or if it is decided that a mastoid operation is necessary the labyrinth should be drained at the same time. The reason for this is the same as that applying in Case 1 above,

[1] Labyrinthitis is said to be rather more common in males than in females, but it is quite fortuitous that all the cases shown were males.

namely, that as the labyrinth is dead we shall get no warning of a possible spread of acute infection (much more probable in a case where the labyrinth is already the site of chronic disease than when the labyrinth is healthy), until meningitis occurs.

**Case 2.**—Chronic pan-labyrinthitis, following acute infection.

E. M., male, aged 53, miner.

May, 1921, c.c.o. giddiness for some weeks, now diminishing, following a severe attack of pain and discharge, right ear. The ear appeared to have been the seat of chronic suppuration for years; it was entirely deaf to all tests; all labyrinthine tests were negative. It was decided that no operation ought to be performed.

When should one *not* operate on the labyrinth? In general, when labyrinthine symptoms occur in the course of acute otitis, free drainage of the middle ear should be secured. Many cases will not require any further operation, and in any event it is probably not sound to open the labyrinth at once, assuming that the case has been seen at a reasonably early stage Secondly, as Mr. Sydney Scott puts it, "the surgeon could rarely have any justification for destroying the labyrinth, so long as positive caloric tests were obtainable,"[1] *i.e.* while the labyrinth is alive.

**Case 3.**—Chronic circumscribed labyrinthitis, with granulations about stapes. Radical mastoid operation and topical applications to inner tympanic wall. Relief of all symptoms, except deafness.

**Case 4.**—Chronic circumscribed labyrinthitis, with fistula of external semicircular canal. Radical mastoid operation; only partial relief of vertigo, etc. Prognosis indefinite.

**Case 5.**—Right chronic circumscribed labyrinthitis with unusual symptoms: ? fistula of the superior semicircular canal. Radical mastoid operation, and topical applications: improvement; operation not indicated. The left ear gives the "pseudo-fistula" sign, with a well-healed mastoid cavity.

[1] *Proc. R. Soc. Med.*, 1924.

It will be noticed that in Case 4 the prognosis is in doubt. The patient is only 26, yet his vertigo, etc., quite prevent even the lightest work. Although one should regard with great reluctance the proposal to eviscerate a living labyrinth, circumstances may arise where this must be considered; the risk, while definite, is not prohibitive, and the result may justify a bold course.

**Case 6.**—Chronic progressive labyrinthitis, with fistula of the external semicircular canal; and disabling vertigo. Barany fistula test negative. Operation: relief.

S. P., male, aged 38, tin miner.

June, 1922, c.c.o. deafness and discharge from the right ear; occasional attacks of vertigo and vomiting. A radical mastoid operation was performed in 1916.

The ear showed a sodden, discharging mastoid cavity. After careful local treatment this cleaned up, except in the upper part, where granulations persisted. Auditory bone conduction normal; labyrinth tests positive.

Fistula test: the usual Barany test was negative throughout. Touching the granulations, however, at once produced vertigo, and nystagmus to the left. Ordinarily there was no spontaneous nystagmus. Seen shortly before the labyrinth operation, during a mild attack of vertigo, there was a fine spontaneous nystagmus to the right.

In spite of local improvement, the vertigo got steadily worse. Nausea became of daily occurrence, at first in the morning, then through the day; vomiting became more and more frequent. Vertiginous attacks occurred two or three times a day; he felt as if spinning round to the left (diseased side) and objects seemed to move round to the right; he staggered but did not fall to the left. Several severe attacks overtook him, in which he fell to the *right*; the other sensations were much more violent than usual, but he did not notice whether they were otherwise different. Such attacks may express the rarely seen phenomenon of dysharmony from stimulation of the labyrinth—nearly always, the signs of disturbance due to disease point to dysharmony from depression of the labyrinthine function.

The man had for months been unable to do any work, and was steadily getting worse. Inasmuch as his whole life was becoming unbearably wretched, I put the situation before him, and it was decided to operate.

March, 1924. At the operation I found a fistula fully 2 mm. wide leading into the external semicircular canal. I did a double vestibulotomy, carefully curetting the vestibular contents, and each ampulla in turn; this point is of importance if vertigo immediately after the operation is to be avoided. From the time he recovered from the anæsthetic, he has not had nausea, vomiting, or vertigo; except that for the first three weeks he had slight vertigo—much less than the mildest attacks from which he had suffered—on sudden movement. There was some trouble at first in walking, though at the meeting this had disappeared, and he could walk even with the eyes shut, without difficulty. Facial paralysis came on on the second day, and was complete by the fourth; this is now improving.

The man's entire carriage and expression have altered. He has put on weight, and feels well. His own description is: "I am quite my old self again."

---

